# Evaluation of the clinical application effect of eSource record tools for clinical research

**DOI:** 10.1186/s12911-022-01824-7

**Published:** 2022-04-11

**Authors:** Bin Wang, Xinbao Hao, Xiaoyan Yan, Junkai Lai, Feifei Jin, Xiwen Liao, Hongju Xie, Chen Yao

**Affiliations:** 1 Peking University Clinical Research Institute, Peking University First Hospital, Beijing, China; 2 Department of Hematology, First Affiliated Hospital of Hainan Medical College, Haikou, China; 3 Boao Lecheng Tigermed Clinical Research Center, Qionghai, China; 4 National Center for Trauma Medicine, Peking University People’s Hospital, Beijing, China; 5 Department of Plastic and Cosmetic Surgery, The Second Affiliated Hospital of Hainan Medical University, Haikou, China; 6 Hainan Institute of Real World Data, Qionghai, China

**Keywords:** Electronic medical record, eSource, Source data, Real-world study, Interoperability, Data collection, Data transcription, System usability scale

## Abstract

**Background:**

Electronic sources (eSources) can improve data quality and reduce clinical trial costs. Our team has developed an innovative eSource record (ESR) system in China. This study aims to evaluate the efficiency, quality, and system performance of the ESR system in data collection and data transcription.

**Methods:**

The study used time efficiency and data transcription accuracy indicators to compare the eSource and non-eSource data collection workflows in a real-world study (RWS). The two processes are traditional data collection and manual transcription (the non-eSource method) and the ESR-based source data collection and electronic transmission (the eSource method). Through the system usability scale (SUS) and other characteristic evaluation scales (system security, system compatibility, record quality), the participants’ experience of using ESR was evaluated.

**Results:**

In terms of the source data collection (the total time required for writing electronic medical records (EMRs)), the ESR system can reduce the time required by 39% on average compared to the EMR system. In terms of data transcription (electronic case report form (eCRF) filling and verification), the ESR can reduce the time required by 80% compared to the non-eSource method (difference: 223 ± 21 s). The ESR accuracy in filling the eCRF field is 96.92%. The SUS score of ESR is 66.9 ± 16.7, which is at the D level and thus very close to the acceptable margin, indicating that optimization work is needed.

**Conclusions:**

This preliminary evaluation shows that in the clinical medical environment, the ESR-based eSource method can improve the efficiency of source data collection and reduce the workload required to complete data transcription.

**Supplementary Information:**

The online version contains supplementary material available at 10.1186/s12911-022-01824-7.

## Background

Electronic sources (eSources) are data that were originally recorded in an electronic format. An eSource generally includes the direct capture, collection, and storage of electronic data (for example, electronic medical records (EMRs), electronic health records (EHRs), or wearable devices) that are used to simplify clinical research [1]. However, eSource can only be possible if the EHRs can support the collection of quality research data. There has been some eSource-related research progress in the field of clinical trials [2–4] and in relatively large projects, such as the OneSource project, EHR4CR project, European FP7 TRANSFoRm project, etc. [5–7]. However, the characteristics of real-world studies (RWSs) requires a large amount of research cost investment for the data collection and quality control, and there are very limited cases and experiences that can be used for reference in this regard.

The ALCOA + (attributable, legible, contemporaneous, original, accurate, complete, consistent, enduring and available) standard has been adopted in good clinical practices (GCP) principles and has become a recognized quality standard for clinical research data [8]. The United States Food and Drug Administration (FDA) pointed out in a recent draft guidance that the EHR system can be modified to collect additional patient data during routine care through additional modules of the EHR system in prospective clinical studies that recommend the use of EHRs [9].

There are more than 300 commercial suppliers of hospital information systems in China, and these systems have various technical structures and data standards [10]. The challenge of applying EMR data to clinical research in China is the lack of data interoperability and the difficulty of extracting free text data. The main highlight is the concern that hospital management departments have regarding the data security [11]. The purpose of clinical research is to solve clinical practice problems, and strengthening the hospital clinical research source data management is the foundation for improving the quality of the clinical research data in China.

In the early work of our research group, a hospital clinical research source data management platform and source data management process architecture were proposed [12]. Subsequently, our research group explored a real-world data (RWD) collection mode based on hospital informatization and verified it using a Catalys Precision Laser System medical device RWS [13]. The CATALYST project completed the registration and marketing approval process in China using the manual data collection method of traditional clinical trials. After the study was completed, the medical data of all subjects was exported by the hospital information department to a technology company for data extraction. We compared the extracted data with the data manually entered into the electronic case report form (eCRF) in the electronic data capture (EDC) system. When natural language processing (NLP) was used, the completion time was reduced by 90% compared to methods that relied on manual input [13]. Our team has explored an integrated eSource solution for hospital real-world data collection, governance and management in many years of clinical research, and we have cooperated with other organizations to develop an innovative eSource record (ESR) system [14]. The goal of this study was to evaluate the efficiency, quality, and system performance of ESR in data collection and data transcription.

## Methods

### System design

**Fig. 1:**
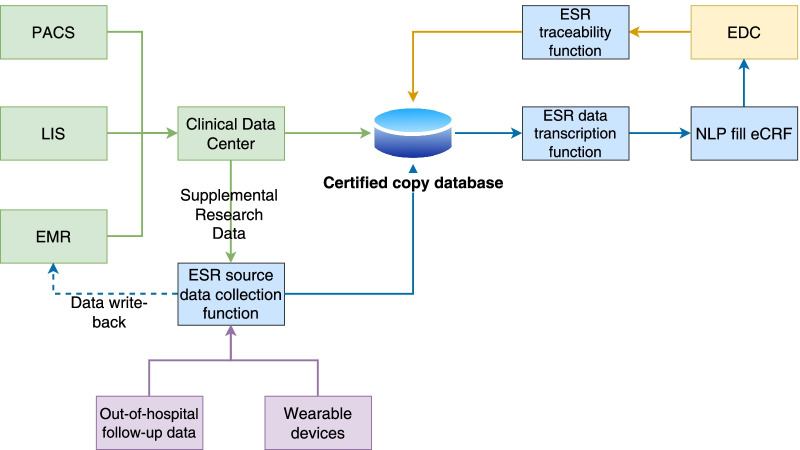
Flow chart of the ESR

The ESR solution includes five steps: research project preparation, initial survey collection, in-hospital medical record writing, out-of-hospital follow-up, and eCRF traceability. Its functions cover the entire clinical research process, and these mainly include the source data collection, data extraction and management, and docking with EDC and health information systems (HISs). Its core concept consists of two steps: integrating the source data from the various sources required for the research to form a certified copy database. The certified copy database will be managed to form a clinical research database. The ESR is designed in accordance with the GCP principle to meet the ALCOA + standard[15] of clinical research data quality and to simultaneously improve the efficiency of clinicians in writing EMRs. ALCOA + is a framework or set of principles that ensures data integrity. It has relevance in a range of areas, particularly in relation to pharmaceutical research, manufacturing, testing, and the supply chain. ESR tools are deployed in hospitals to achieve medical data security. The design framework of the ESR system is shown in Fig. 1.

By connecting EMR templates in the hospital or creating new templates, users can input information without changing their usage habits. Clinicians configure the same medical record form used in the EMR system in the ESR system and record research medical records according to the requirements of the research plan; then, ESR writes the contents of the EMR form back to the EMR system. Because the content of research medical records is larger than that of routine medical records, clinicians choose the content range that needs to be written back. For the out-of-hospital follow-up data collected in the eCRF and the data that cannot be accommodated in the in-hospital EMR form (such as various scale scoring data), no field mapping and docking with the EMR system is performed. In terms of input methods, the ESR system not only supports traditional manual input but also allows clinicians to complete medical records more efficiently through voice input and medical record prefilling functions and collect out-of-hospital data through out-of-hospital follow-up functions, such as official WeChat accounts. In the process of information input, the built-in data verification logic function of the system will scan in real time and ensure data quality by instantly alerting users if incorrect information is recorded in the medical record or research data are missing.

ESR connects the hospital’s laboratory information systems (LIS) and picture archiving and communications systems (PACS) to form a certified copy database of the hospital source data through backup. Out-of-hospital follow-up and EMR source data recorded in the ESR are entered into the certified copy database at the same time. After the medical record is completed, according to the data collection requirements predefined by the research plan, the system can automatically identify the information, extract the research data to the corresponding data elements, and support users in tracing the extraction results. At the same time, the system records and leaves traces of users’ modification operations, including the modified personnel, time, and content, to ensure the traceability of the data.

The ESR system uses NLP to automatically extract the data from the certified copy database in real time and to enter it into the eCRF, and it also supports the traceability and viewing of the source data. The clinical research coordinator (CRC) does not need to manually fill in the eCRF, but only the eCRF traceable verification work is performed in the ESR. Through the interface between the ESR and EDC, the eCRF data submitted by the CRC are transmitted to the EDC to form a mirrored eCRF. Through the traceability interface developed in the EDC, the clinical research associate (CRA) performs the routine source data verification and query work and sends the query to the ESR through the interface to remind clinicians to correct the medical record.

### Research design

This is a single-center observational study. Participants needed to use the eSource method and the non-eSource method to complete two workflows. The goal of this study is to evaluate the impact of the two processes on source data collection (EMR writing) and data transcription (eCRF filling and verification). The collected research data include the time spent and the eCRF accuracy rate. A stopwatch was used for timing, and the time was manually entered into the Excel table. The total time for recording EMRs included three parts: the collection of the medical history, the entering of basic information, and the writing of the medical record.

The workflow of the traditional data collection and manual transcription (non-eSource method): the clinicians use the keyboard to manually input the data into the EMR system to record the medical records, and the CRC manually fills in the eCRF and checks the data based on the source data of the EMRs.

The ESR-based source data collection and electronic transmission (eSource method) workflow: the same medical record form is used in the ESR as is used in the EMR, clinicians use the ESR to record the medical records, and the ESR provides voice recognition, optical character recognition (OCR), picture recognition, dialog recording and NLP intelligent filling of the medical records and other functions. The ESR uses NLP to automatically extract data from the EMRs in a text form and is used to fill in the eCRF. When checking the source, the CRC needs to check the correctness of the fields that were filled in by the NLP system and need to manually correct the incorrectly entered fields. The field composition, data types and recording methods of outpatient medical records in the ESR system are shown in Table 1. The data sources of the eCRF data variables and the extraction methods using the ESR system are shown in Table 2.

**Table 1:** Field composition, data type and recording method of outpatient medical records in the ESR system

Field name	Data type	Main recording method
Demographics	Structured	Optical character recognition
Visit time	Structured	Voice input
History of present illness	Free text	Voice recognition/NLP parsing transcribed text to generate records
Past history	Free text	Voice recognition/NLP parsing transcribed text to generate records
Personal history	Free text	Voice recognition/NLP parsing transcribed text to generate records
Menstrual history	Free text	Voice recognition/NLP parsing transcribed text to generate records
Marriage history	Free text	Voice recognition/NLP parsing transcribed text to generate records
Physical examination	Free text	Voice input
Specialty situation	Free text	Voice input
Auxiliary examination	Free text	Voice input/optical character recognition
Initial diagnosis	Structured	Voice input
Treatment plan	Free text	Voice input

**Table 2:** Data sources for eCRF data variables and extraction methods using the ESR system

Research variable	Data sources	Source data record type	Extraction method
Subject information	Automatically generated by ESR	Structured	Field mapping
Date of visit	Outpatient medical records (visit time)	Structured	Field mapping
Signature of informed consent	Electronic informed consent/dialogue recording	Structured/sound	Field mapping
Population statistics	Outpatient medical records (demographics)	Structured	Field mapping
Facial aesthetic treatment	Outpatient medical records (past history)/dialogue recording	Free text/sound	Natural language processing
Vital signs	Outpatient medical records (physical examination)	Free text	Natural language processing
Physical examination	Outpatient medical records (physical examination)	Free text	Natural language processing
Digital photographs	Patient photo metadata	Image	Field mapping
Inclusion criteria	eCRF (inclusion criteria)	Structured	Clinician fills in manually in eCRF
Exclusion criteria	eCRF (exclusion criteria)	Structured	Clinician fills in manually in eCRF
Preliminary screening conclusion	Outpatient medical records (treatment plan)	Free text	Natural language processing
Final screening conclusion	Outpatient medical records (treatment plan)	Free text	Natural language processing
Past/concomitant drug therapy	Outpatient medical records (past history)/dialogue recording	Free text/sound	Natural language processing
Concomitant nondrug therapy	Outpatient medical records (past history)/dialogue recording	Free text/sound	Natural language processing

### Implementation process

We selected an RWS to evaluate the effectiveness and safety of beauty medical equipment (cross-linked glucan) for chin augmentation in the Boao Lecheng pilot zone. This study was designed as a prospective, single-center, observational study. Considering that the research data provided in the out-of-hospital follow-up and surgical records are relatively structured, we selected the form of outpatient medical records that were represented by a highly free text record form to evaluate the two workflows. At the beginning of the project, all participants were trained on the program and the ESR tools. The eCRF of the screening visit of this RWS used in our study is provided in Additional file 1.

The beta version of the ESR was deployed in a medical institution in June 2021. To avoid affecting the normal diagnosis and treatment within the department and to allow clinicians to gradually adapt to the new data collection method, we chose the first two months as a transition period to complete the docking and customized work. In June and July 2021, only the time that was spent by the clinicians completing the EMRs using the traditional methods was counted, and the eCRF entry tasks were not performed. Starting in August 2021, the ESR system was officially used to replace the previous writing method that was used for the EMRs. The CRC used both the eSource and non-eSource methods. A total of 4 clinicians and 14 experienced experts from contract research organization (CRO) companies (2 CRA, 5 CRC, 2 data manager (DM), 4 project manager (PM)) participated in this test task. We collected feedback and suggestions from all users, discussed any issues and summarized the experience in the form of a web meeting, which provided insights and a basis for the design and optimization of subsequent ESRs, and we sent an invitation link to a rating scale to evaluate the performance of the ESRs.

### Rating scale

**Fig. 2:**
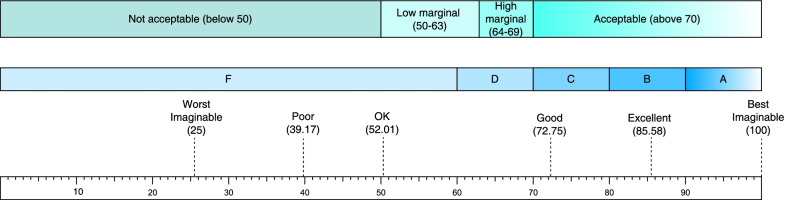
The SUS scoring standard. (Adapted from Bangor et al. [17, 18])

The system usability scale (SUS) [16] was created by John Brooke in the 1980s and has been used in more than 1,500 studies in multiple industries. It is the industry standard for usability research. The SUS ranges from 0 (worst) to 100 (best). The cross-industry average system availability scale score is 68, so this value is considered the threshold of acceptable availability. There are also acceptable ranges and grading scales to explain the SUS scores (Fig. 2, adapted from Bangor et al. [17, 18]). The SUS is a survey consisting of 10 questions using a 5-point Likert scale ranging from “strongly disagree” to “strongly agree”, with a score of 1–5 for each question [19]. The odd-numbered questions were positive, such as “I felt very confident using the system”, and the even-numbered questions were negative, such as “I found the system very cumbersome to use”. The conversion method was as follows: 1 was subtracted from the user’s score for odd-numbered items. For the even-numbered items, the user rating was subtracted from 5. This scaled all of the values from 0 to 4 (4 was the most positive response). Each of the user’s conversion score was added up and was multiplied by 2.5. This converted the range of possible values from 0 to 100.

The system applicability and record quality are also important factors that affect the acceptance of the ESR system by clinicians or users such as the CRC. We chose the questionnaire items based on the items on the EHR scale that were used in Salleh et al. [20]. The questionnaire designed by Salleh et al. contains six subcategories. After consulting with statisticians, clinicians, information experts and other experts, we decided to select only 2 subcategories (system quality and record quality) according to the purposes of this study. The system quality entry contains 4 components (adequate IT infrastructure, system interoperability, system security, and system compatibility). After comparison with the SUS score, we found that the system interoperability entry with only 3 questions was not sufficient to assess system usability, so it was not adopted. The adequate IT infrastructure item was not suitable for ESR systems and therefore was not selected. Therefore, the overall questionnaire has 3 parts: the participant information, the SUS evaluation of the ESR system, and the evaluation of the other characteristics of the ESR system (system security, system compatibility, and record quality). Since the components of the original questionnaire by Salleh et al. are relatively independent, we screened them only according to the needs of this research and did not modify the content of the questionnaire. Therefore, the survey results are still valid and reliable.

### Data analysis

Mann-Whitney U test was used for the statistical comparisons. The data analysis software used in this study was Python (version 3.7.11). In all of the analyses, a two-sided p < 0.05 was considered statistically significant.

## Results

### Data collection

The research data of all of the enrolled participants were collected from June to October 2021. The participants were the patients enrolled in the RWS study. All the participants enrolled in that period were included in our study. A total of 90 participants were enrolled, including 19 participants in June, 9 in July, 0 in August (due to epidemic control reasons), 18 in September, and 44 in October.

### Evaluation of the efficiency of writing the EMRs

**Fig. 3:**
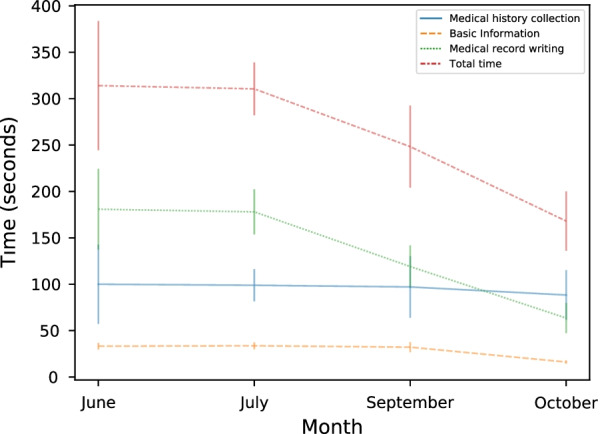
The time spent on EMR records for each patient in the different months. The error bars in the figure were drawn based on the mean and standard deviation

A total of 28 EMRs were completed in June and July 2021 using the EMR system, and a total of 62 EMRs were completed in September and October using the ESR system. The change trend of the EMR recording the average time spent for each patient in the different months is shown in Fig. 3. Compared with the traditional keyboard input method used in the EMR system, the various additional functions of the ESR (such as voice recognition, OCR recognition, etc.) allowed for less time spent filling in the basic information and writing the medical records, and during the medical history collection, there was no change. In terms of the total time, the ESR system can reduce the required time by an average of 39%. The results of the comparison between the two groups are shown in Table 3.

**Table 3:** The time spent on EMR records for each patient using the different systems (unit: seconds)

Item	Total	EMR system	ESR	P-value^a^
N	90	28	62	
Medical history collection, mean (SD)	93.6 (30.5)	99.6 (35.4)	90.9 (28.0)	0.255
Basic information, mean (SD)	24.7 (8.8)	33.4 (2.7)	20.8 (7.8)	< 0.001
Medical record writing, mean (SD)	110.8 (57.0)	179.9 (37.1)	79.6 (30.8)	< 0.001
Total time, mean (SD)	229.2 (77.3)	312.9 (58.1)	191.3 (50.6)	< 0.001

^a^Mann–Whitney U test

### eCRF data transcription time

Since no eCRF data transcription work was performed in June and July 2021, data from a total of 62 of the patients were used for comparison. The eCRF corresponding to each of the patient’s outpatient medical record form had 33 fields, so there were a total of 2046 fields. The difference between using the eSource (55 ± 12 s) and non-eSource methods (277 ± 19 s) was statistically significant (p < 0.001, paired t test). The eSource method can reduce the required time by 80% (difference: 223 ± 21 s).

### eCRF data transcription quality

In the non-eSource methods, the overall correct rate of the CRC’s first entry was 93.79%. After manual data verification by CRA, the final research data were 100% accurate. The CRC’s main input error fields were concentrated in filling in various numerical values with decimal places. In the eSource method, the CRC found that the overall NLP extraction accuracy was 96.92% when checking the original research data for NLP extraction. For the fields wrongly extracted by NLP, the CRC supplemented the input; the final research data accuracy rate was also 100%. The fields with errors filled in using the eSource method extracted by NLP are mainly fields such as “previous beauty history” and date and time data. In the original medical records recorded by clinicians, there are fields with wrong source data, mainly numerical values, date data, and spelling errors. The data quality of the two methods is compared in Table 4.

**Table 4:** Data quality comparison between the two methods

Month	Total number of eCRF fields	Non-eSource method	eSource method	
		Correct fields entered for the first time in CRC, n (%)	NLP fills in correct fields, n (%)	CRC correction fields, n (%)
September	594	560 (94.28)	567 (95.45)	27 (4.55)
October	1452	1359 (93.60)	1416 (97.52)	36 (2.48)
Total	2046	1919 (93.79)	1983 (96.92)	63 (3.08)

### The ESR performance evaluation questionnaire

The questionnaire invitation link was sent in the form of an e-mail to 18 people who participated in the project. A total of 13 questionnaires were received, with a response rate of 72%. The characteristics of the population participating in the questionnaire survey are shown in Table 5.

**Table 5:** The characteristics of the population participating in the questionnaire survey

Items		Total	CRO experts	Clinicians
n		13	9	4
Gender, n (%)	Female	10 (76.9)	9 (100.0)	1 (25.0)
	Male	3 (23.1)		3 (75.0)
Age, mean (SD)		29.2 (3.4)	29.3 (4.0)	29.0 (2.2)
Profession, n (%)	CRC	2 (15.4)	2 (22.2)	
	CRC/PM	2 (15.4)	2 (22.2)	
	DM	1 (7.7)	1 (11.1)	
	PM	4 (30.8)	4 (44.4)	
	Clinicians	4 (30.8)		4 (100.0)
Highest education, n (%)	PhD student	1 (7.7)		1 (25.0)
	College degree and below	1 (7.7)	1 (11.1)	
	Undergraduate	8 (61.5)	5 (55.6)	3 (75.0)
	Postgraduate	3 (23.1)	3 (33.3)	
Experience in the medical field, n (%)	1–3 years	5 (38.5)	1 (11.1)	4 (100.0)
	4–6 years	2 (15.4)	2 (22.2)	
	7–9 years	6 (46.2)	6 (66.7)	
Frequency of using the EMR system, n (%)	Not applicable	9 (69.2)	9 (100.0)	
	Regularly	1 (7.7)		1 (25.0)
	Use every day	2 (15.4)		2 (50.0)
	Frequently used	1 (7.7)		1 (25.0)
Frequency of using the EDC system, n (%)	Not applicable	7 (53.8)	3 (33.3)	4 (100.0)
	Sometimes	2 (15.4)	2 (22.2)	
	Never	1 (7.7)	1 (11.1)	
	Daily	1 (7.7)	1 (11.1)	
	Often	2 (15.4)	2 (22.2)	

In terms of usability, the average overall SUS score of the ESR was 66.9 points, and the median was 70 points. The classification of the SUS belongs to the D level. The results of the ESR’s SUS evaluation are shown in Table 6. Compared to the 68-point threshold, the ESR was very close to the acceptable margin, indicating that subsequent system optimization work is needed. In terms of the system security, system applicability, and recording quality, the average value of all the scoring items in the ESR was 4 points or more, indicating that the participants gave a good evaluation for these performance characteristics of the ESR. The evaluation results of the other performance characteristics of the ESR are shown in Table 7.

**Table 6:** The SUS score of the ESR

Items^a^	Total	CRO experts	Clinicians
n	13	9	4
1. I think that I would like to use this system frequently			
Mean (SD)	4.1 (1.2)	3.8 (1.3)	4.8 (0.5)
Median [Q1,Q3]	4.0 [4.0,5.0]	4.0 [3.0,5.0]	5.0 [4.8,5.0]
2. I found the system unnecessarily complex			
Mean (SD)	2.5 (1.1)	2.9 (1.1)	1.8 (1.0)
Median [Q1,Q3]	2.0 [2.0,3.0]	3.0 [2.0,3.0]	1.5 [1.0,2.2]
3. I thought the system was easy to use			
Mean (SD)	4.3 (0.9)	4.3 (0.5)	4.2 (1.5)
Median [Q1,Q3]	4.0 [4.0,5.0]	4.0 [4.0,5.0]	5.0 [4.2,5.0]
4. I think that I would need the support of a technical person to be able to use this system			
Mean (SD)	3.5 (1.3)	3.3 (1.4)	3.8 (1.3)
Median [Q1,Q3]	4.0 [2.0,4.0]	4.0 [2.0,4.0]	4.0 [3.5,4.2]
5. I found the various functions in this system were well integrated			
Mean (SD)	3.9 (0.8)	3.9 (0.8)	4.0 (0.8)
Median [Q1,Q3]	4.0 [3.0,4.0]	4.0 [3.0,4.0]	4.0 [3.8,4.2]
6. I thought there was too much inconsistency in this system			
Mean (SD)	2.8 (1.0)	2.9 (1.1)	2.8 (1.0)
Median [Q1,Q3]	3.0 [2.0,3.0]	3.0 [2.0,3.0]	2.5 [2.0,3.2]
7. I believe that most people would learn to use this system very quickly			
Mean (SD)	4.3 (0.6)	4.1 (0.6)	4.8 (0.5)
Median [Q1,Q3]	4.0 [4.0,5.0]	4.0 [4.0,4.0]	5.0 [4.8,5.0]
8. I found the system very cumbersome to use			
Mean (SD)	2.5 (1.1)	2.7 (1.1)	2.0 (0.8)
Median [Q1,Q3]	2.0 [2.0,3.0]	2.0 [2.0,3.0]	2.0 [1.8,2.2]
9. I felt very confident using the system			
Mean (SD)	4.2 (0.8)	4.1 (0.8)	4.5 (1.0)
Median [Q1,Q3]	4.0 [4.0,5.0]	4.0 [4.0,5.0]	5.0 [4.5,5.0]
10. I needed to learn many things before I could start using this system			
Mean (SD)	2.8 (1.2)	3.1 (1.2)	2.0 (0.8)
Median [Q1,Q3]	3.0 [2.0,3.0]	3.0 [2.0,3.0]	2.0 [1.8,2.2]
**The SUS total score**^b^			
Mean (SD)	66.9 (16.7)	63.3 (15.4)	75.0 (18.8)
Median [Q1,Q3]	70.0 [50.0,80.0]	60.0 [50.0,75.0]	81.2 [71.9,84.4]

^a^The 10 items of the SUS are calculated using the unconverted raw scores

^b^The SUS total score needs to be converted

**Table 7:** Evaluation of the other performance characteristics of the ESR system

Items	Total	CRO experts	Clinicians
n	13	9	4
**System security**			
1. I believe that the system does not allow for unauthorized access			
Mean (SD)	4.4 (1.1)	4.2 (1.3)	4.8 (0.5)
Median [Q1,Q3]	5.0 [4.0,5.0]	5.0 [4.0,5.0]	5.0 [4.8,5.0]
2. I believe the system protects the patient’s information			
Mean (SD)	4.5 (0.5)	4.4 (0.5)	4.8 (0.5)
Median [Q1,Q3]	5.0 [4.0,5.0]	4.0 [4.0,5.0]	5.0 [4.8,5.0]
3. I believe the system has a robust security control mechanism			
Mean (SD)	4.5 (0.7)	4.4 (0.5)	4.5 (1.0)
Median [Q1,Q3]	5.0 [4.0,5.0]	4.0 [4.0,5.0]	5.0 [4.5,5.0]
4. I feel secure and safe using the EHR system			
Mean (SD)	4.5 (0.7)	4.3 (0.7)	4.8 (0.5)
Median [Q1,Q3]	5.0 [4.0,5.0]	4.0 [4.0,5.0]	5.0 [4.8,5.0]
**System compatibility**			
1. The system fits my workflows			
Mean (SD)	4.2 (0.7)	4.2 (0.7)	4.0 (0.8)
Median [Q1,Q3]	4.0 [4.0,5.0]	4.0 [4.0,5.0]	4.0 [3.8,4.2]
2. The system fits the way I work and my work styles			
Mean (SD)	4.4 (0.7)	4.2 (0.7)	4.8 (0.5)
Median [Q1,Q3]	4.0 [4.0,5.0]	4.0 [4.0,5.0]	5.0 [4.8,5.0]
3. The system fits my clinical practices			
Mean (SD)	4.2 (0.7)	4.1 (0.8)	4.5 (0.6)
Median [Q1,Q3]	4.0 [4.0,5.0]	4.0 [4.0,5.0]	4.5 [4.0,5.0]
4. The system fits my patients’ needs			
Mean (SD)	4.0 (0.7)	4.2 (0.7)	3.5 (0.6)
Median [Q1,Q3]	4.0 [4.0,4.0]	4.0 [4.0,5.0]	3.5 [3.0,4.0]
**Records quality**			
1. The information output is timely and up-to-date			
Mean (SD)	4.5 (0.7)	4.2 (0.7)	5.0 (0.0)
Median [Q1,Q3]	5.0 [4.0,5.0]	4.0 [4.0,5.0]	5.0 [5.0,5.0]
2. The system is consistent when viewing it from other computers			
Mean (SD)	4.3 (0.8)	4.0 (0.7)	5.0 (0.0)
Median [Q1,Q3]	4.0 [4.0,5.0]	4.0 [4.0,4.0]	5.0 [5.0,5.0]
3. The system is available in a standardized format			
Mean (SD)	4.4 (0.7)	4.2 (0.7)	4.8 (0.5)
Median [Q1,Q3]	4.0 [4.0,5.0]	4.0 [4.0,5.0]	5.0 [4.8,5.0]
4. The information output is accurate and reliable			
Mean (SD)	4.4 (0.7)	4.1 (0.6)	5.0 (0.0)
Median [Q1,Q3]	4.0 [4.0,5.0]	4.0 [4.0,4.0]	5.0 [5.0,5.0]
5. The information output is complete			
Mean (SD)	4.5 (0.7)	4.2 (0.7)	5.0 (0.0)
Median [Q1,Q3]	5.0 [4.0,5.0]	4.0 [4.0,5.0]	5.0 [5.0,5.0]

## Discussion

Compared with the traditional way of recording EMRs by using keyboard input, our research shows that the assistance of voice recognition can have a positive impact, which is consistent with the conclusions of previous studies [21, 22]. Compared with clinicians who use the traditional method of using the keyboard to quickly record the main points of the medical history in the EMR system during the consultation process, there are more recognition errors due to the patient’s accent during the ESR voice consultation and recognition process. Clinicians need to use their voice to actively retell or summarize the main points, so the two processes have no difference in the medical history collection part. Nevertheless, the recording function of the consultation provided by ESR can help doctors trace back the consultation discussion at any time, thus allowing for the timely collection of source data and avoiding the mistakes introduced by the recall after the consultation.

In terms of the eCRF data transcription, compared with the 90% time cost savings that was determined in the previous ophthalmology project, the 80% time savings results of our research also demonstrates the obvious advantages of the eSource process. The study by Nordo et al. found that eSource can save 37% of the time required in the clinical registration and data collection [23]. The decision analysis model of Eisenstein et al. estimated that the cost of the CRC data collection in clinical trials can be reduced by $68 per patient [24]. A potential problem of using NLP to implement eSource is that the accuracy of model extraction is easily affected by the standardization of medical records. The study by Velupillai et al. outlines the operability recommendations for the application of NLP methods in the clinical field [25]. Although we provided a medical record template in the ESR to promote the standardization of medical records, we found that in some fields, such as “previous beauty history”, had more extraction errors. This is because at the beginning of the study, we only used 30 corpora to train the basic NLP model. With the accumulation of more medical records, the term dictionary can be expanded, and the recognition effect will be improved. Because a manual standard corpus is required to train the basic model, the ESR that includes only a small amount of corpus reduces the labor cost of research project preparation.

In terms of the previous EHR usability assessments, a study of 15 EHR systems in the UK found that the median SUS score was 53 (IQR 35–68) [26]. In a study of 870 doctors in 18 medical majors in the United States, the mean SUS score was 45.9 ± 21.9 [27]. The lowest median SUS score obtained in our study was 60 (IQR 50–75) when evaluated by the CRO experts, which was higher than the results of previous studies. Combining the SUS scores given by clinicians and the quantitative evaluation results on the time spent on the EMRs, we can conclude that ESR can improve the efficiency of clinicians and is easily accepted. The sample size has nothing to do with the reliability, so the SUS can be used with very small sample sizes (as few as two users) and still produce reliable results.

Based on our practical experience, one of the challenges of implementing eSource is the difference between free text and structured input. A review by Forsvik et al. [28] mentioned that narrative text is the most difficult to replace when describing the thought process, and it may be beneficial to merge the two data types. Allowing the input of free text and structured text may increase the user acceptance [29]. Busy clinicians usually value flexibility and efficiency, while those clinicians who reuse data usually value structure and standardization. The study by Rosenbloom et al. elaborated on the tension between structured and free text [30]. Unstructured, structured and coded data need not be mutually exclusive, and a hybrid model called semistructured data has been suggested in the literature [31]. Therefore, the input of semistructured data may help balance the contradiction between the efficiency of clinicians’ medical record writing and the accuracy of NLP extraction.

In the process of implementing eSource, other lessons that need to be learned include the following: when integrating data from multiple sources, one-click linking to the real data source should be implemented. Reducing the verification of multiple systems or documents that are considered to be the data sources and improving the accuracy and efficiency are expected to benefit the field of clinical research. Compared with the traceability of EMRs, the traceability of adverse events and combined medications is more difficult because these source data are from different places and this type of data have more sources. Therefore, the management and integration of these diversified electronic source data has brought great challenges.

Although the SUS score shows that ESR is close to acceptable, it still needs to be upgraded according to the experience of different users in the later promotion. For clinicians, the problems that need to be solved include the following: (1) A mobile application should be developed. Considering the portability of mobile phones and the convenience of recording, it is recommended that the system support mobile phone recording and photo uploading as well as OCR recognition to collect raw data more efficiently. (2) To broaden the application scenarios of OCR image recognition, in addition to demographic information and laboratory examination, various inspection reports such as other paper medical records should be considered. (3) The degree of interaction with the EMR system should be increased so that clinicians can avoid frequently switching back and forth between systems during use and so that the use process is smoother. For CRO experts, the following suggestions are made: (1) Mainly solve certain special scenarios with challenges in traceability, such as the traceability of unplanned visits, adverse events and the concomitant drugs. (2) Consider compliance issues, such as whether the regulatory authorities will accept this new method of data extraction and traceability when conducting on-site inspections and how to communicate during the project review process by regulatory authorities. (3) Add automatic reminders for adverse events: laboratory inspection values should ​​have corresponding normal value ranges, and a logical correlation and verification function should be added to realize the reminder function for adverse event entry.

Our research has some limitations. As with all research involving surveys, the likelihood of response deviation and the representativeness of the samples are important issues. However, because this study is based on a real research project, the surveyed personnel can only be limited to all of the research members participating in this project. Second, our research objectives are mainly focused on the feasibility evaluation of eSource, so we only evaluated the performance of the ESR system in an important part of the research process. It is foreseeable that ESR is expected to reduce the labor costs based on its effect on the entire process of the project. In terms of the scalability, although it this study was only based on a single-center evaluation of a project, we are also implementing multiple RWS projects in other hospitals. In addition, we are also conducting in-depth cooperation with EMR manufacturers to promote the integration of ESRs in EMR systems.

## Conclusion

This preliminary evaluation of the application effect of the ESR system in the clinical medical environment shows that the tool can improve the efficiency of the source data collection and can reduce the workload required to complete the data transcription. The ESR system is designed based on the GCP standard of data quality control and traceability. The built-in NLP can flexibly deal with the extraction of text data, and this provides a new strategy for the realization of the eSource process. However, further research is needed in a different context to verify our findings.

## Supplementary Information

**Additional file 1.** Supplementary Information.

## Data Availability

The datasets used and/or analyzed during the current study are available from the corresponding author on reasonable request.

## Abbreviations

ESR: ESource record
EMRs: Electronic medical records
RWS: Real-world study
RWD: Real-world data
EHRs: Electronic health records
CRO: Contract research organization
EDC: Electronic data capture
eCRF: Electronic case report form
FDA: The United States Food and Drug Administration
GCP: Good clinical practices
OCR: Optical character recognition
CRC: Clinical research coordinator
CRA: Clinical research associate
DM: Data manager
PM: Project manager
NLP: Natural language processing
ALCOA +: Attributable, legible, contemporaneous, original, accurate, complete, consistent, enduring and available
PACS: Picture archiving and communications systems
LIS: Laboratory information systems
HISs: Health information systems
SUS: System usability scale
