# Supplementary material for: Evaluation of the clinical application effect of eSource record tools for clinical research
Source: BMC Med Inform Decis Mak. 2022 Apr 11;22:98. doi: 10.1186/s12911-022-01824-7 (PMC9004184; doi:10.1186/s12911-022-01824-7)
Supplement: Supplementary file 1 — Additional file 1. Supplementary Information. [file 12911_2022_1824_MOESM1_ESM.docx]

**Case Report Form**

| **SUBJECT INFORMATION** |
| --- |
| Subject No.：\|S\|__\|__\|__\|< Automatic Recode > |

| **DATE OF VISIT** |
| --- |
| Date of visit：\|__\|__\|__\|__\|/\|__\|__\|/\|__\|__\| (YYYY-MM-DD) |

| **SIGNATURE OF INFORMED CONSENT** |
| --- |
| Whether the subject has accepted the interpretation of the research process and signed the informed consent？  □**_1_** Yes □**_2_** No |
| Date of signature： \|__\|__\|__\|__\|/\|__\|__\|/\|__\|__\| (YYYY-MM-DD) |

| **POPULATION STATISTICS** | |
| --- | --- |
| Date of birth： | \|__\|__\|__\|__\|/\|__\|__\|/\|__\|__\| (YYYY-MM-DD) |
| Age： | \|__\|__\| (Years)< Automatic Recode > |
| Gender： | □**_1_** Male □**_2_** Female |
| Ethnic origin： | □**_1_** The Han nationality □**_2_** Other |
| If choose “other”, please specify：______ | |

| **FACIAL AESTHETIC TREATMENT** | | | |
| --- | --- | --- | --- |
| Whether the subject has a history of any prior facial aesthetic treatment from 12 months prior to screening？ □**_1_** Yes □**_2_** No | | | |
| **If yes, please provide the following information (multiple choices)** | | | |
| No. | Type of facial aesthetic treatment | Treatment region | Date of facial aesthetic treatment  (YYYY-MM-DD) |
| 1 |  |  |  |
| 2 |  |  |  |
| 3 |  |  |  |

**Logline（add a new logline）**

**Type of facial aesthetic treatment： 1=Botulinum toxin; 2=HA filler; 3=Autologous fat; 4=Thread lifting; 5= Laser or chemical exfoliation; 6=Cosmetic facial surgery; 7=Other, please specify：______**

| **VITAL SIGNS** | |
| --- | --- |
| Does the subject perform vital sign measurements? □**_1_** Yes □**_2_** No  If no, please specify：______ | |
| Examine date：\|__\|__\|__\|__\|/\|__\|__\|/\|__\|__\| (YYYY-MM-DD) | |
| Body temperature measurement site: □**_1_** Armpit □**_2_** Forehead □**_2_** Ear | |
| Body temperature (°C) | \|__\|__\|.\|__\| |
| Heart rate (Times/Min) | \|__\|__\|__\| |
| SBP（mmHg） | \|__\|__\|__\| |
| DBP（mmHg） | \|__\|__\|__\| |
| Respiration rate (Times/Min) | \|__\|__\| |

**Note：If screening and treatment are performed on the same day, vital signs will be measured and recorded only once prior to randomization.**

| **PHYSICAL EXAMINATION** | | |
| --- | --- | --- |
| Does the subject perform physical examination？ □**_1_** Yes □**_2_** No  If no, please specify：______ | | |
| Examine date：\|__\|__\|__\|__\|/\|__\|__\|/\|__\|__\| (YYYY-MM-DD) | | |
| Weight：\|__\|__\|__\|.\|__\|kg | | |
| BMI：\|__\|__\|__\| kg/m^2 < Automatic Recode > | | |
| **Examine item** | **Clinical assessment*** | **If choose abnormal, please specify** |
| Skin and mucous |  |  |
| Lymph nodes |  |  |
| Head (eyes, nose, ears, mouth) |  |  |
| Neck (thyroid, vessels, trachea) |  |  |
| Chest (heart, lungs) |  |  |
| Abdomen (liver, spleen, kidney) |  |  |
| Muscle-skeleton |  |  |
| Neurologic systems |  |  |

**Clinical assessment：1=Normal；2=Abnormal but clinically insignificant ；3= Abnormal and clinically significant；4=** **No measurement result**

| **DIGITAL PHOTOGRAPHS** | | | | |
| --- | --- | --- | --- | --- |
| Was the subject photographed? □**_1_** Yes □**_2_** No | | | | |
| If no, please specify：__________________ | | | | |
| No. | Photo shoot date | Photo shoot start time | Photo shoot end time | Number of photos |
| 1 | \|__\|__\|__\|__\|/\|__\|__\|/\|__\|__\| | \|__\|__\|：\|__\|__\| | \|__\|__\|：\|__\|__\| | \|__\| |
| 2 |  |  |  |  |
| 3 |  |  |  |  |

| **INCLUSION CRITERIA** | |
| --- | --- |
| **Does the subject meet all the criteria for inclusion？** | □**_1_** Yes  □**_2_** No |
| **If no, please check the unmatched inclusion criteria.** | |
| （1）Men or women aged ≥18 years. Subjects seeking for chin volume augmentation. |  |
| （2）Per Investigator’s assessment, subjects who require 1 to 3 mL total volume of cross-linked glucan for chin augmentation to achieve a meaningful improvement and/or change in their aesthetic appearance. |  |
| （3）Subjects who voluntarily decided the participation of the study and signed the informed consent. |  |

**Note: If the answer to any of the inclusion criteria is selected, then this subject cannot participate in this study).**

| **EXCLUSION CRITERIA** | |
| --- | --- |
| **Does the subject not meet all the criteria for exclusion？** | □**_1_** Yes  □**_2_** No |
| **If no, please check the matched exclusion criteria.** | |
| （1）Subjects who are contraindicated to injection with product. |  |
| （2）Other conditions the Investigator considers inappropriate for enrollment. |  |

**Note: If the answer to any of the exclusion criteria is selected, then this subject cannot participate in this study.**

| **PRELIMINARY SCREENING CONCLUSION** |
| --- |
| Subject was preliminary screened successfully or not？ □**_1_** Yes □**_2_** No |
| **If no, please detailed record screening failure information：** |
| The reason for screening failure, only select the most important one： |
| □**_1_** The subject did not meet the inclusion criteria/meet exclusion criteria, please specify |
| □**_2_** Subject withdraw informed consent |
| □**_3_** Adverse events |
| □**_4_** Subject lost to follow-up |
| □**_5_** Other, please specify |
| If choose “Did not meet the inclusion criteria/meet exclusion criteria”, please specify：_________________  If choose “Other”, please specify:__________________ |

| **FINALLY SCREENING CONCLUSION** |
| --- |
| Subject was finally screened successfully or not？ □**_1_** Yes □**_2_** No |
| **If no, please detailed record screening failure information：** |
| The reason for screening failure, only select the most important one： |
| □**_1_** The subject did not meet the inclusion criteria/meet exclusion criteria, please specify |
| □**_2_** Subject withdraw informed consent |
| □**_3_** Adverse events |
| □**_4_** Subject lost to follow-up |
| □**_5_** Other, please specify |
| If choose “Did not meet the inclusion criteria/meet exclusion criteria”, please specify：_________________  If choose “Other”, please specify:__________________ |
| Date of inclusion: \|__\|__\|__\|__\|/\|__\|__\|/\|__\|__\|(YYYY-MM-DD) |
| Whether the subject is a run-in subject? □**_1_** Yes □**_2_** No |

**Past/Concomitant Drug therapy**

| **PAST/CONCOMITANT DRUG THERAPY** |
| --- |
| Did the subjects receive drug therapy within 3 months before screening and during the study？□**_1_** Yes □**_2_** No |
| **If yes, please provide the following information：** |
| Generic name：___________ |
| Unit**^1^**：_______ |
| Frequency**^2^**：__________ |
| Dose per administration：__________ |
| Route of administration**^3^**：_________ |
| Start date：\|__\|__\|__\|__\|/\|__\|__\|/\|__\|__\| (YYYY-MM-DD)  Start time(if applicable)：\|__\|__\|：\|__\|__\|（HH：MM） |
| Ending date：\|__\|__\|__\|__\|/\|__\|__\|/\|__\|__\|(YYYY-MM-DD)  Ending time(if applicable)：\|__\|__\|：\|__\|__\|（HH：MM） |
| Still use or not：□**_1_** Yes □**_2_** No |
| Purpose of treatment^4^：___________ |
| Indication：___________ |

**Logline （add a new logline）**

**1. Units: 1 = mg; 2 = microgram; 3 = milliliter; 4 = U; 5-drop; 6 = tablet; 7 = capsule; 8 = unknown; 9 = other,____**

**2. Frequency: 1=QD once daily; 2=BID twice daily; 3=TID daily 3 times; 4=QOD every other day; 5=PRN on demand; 6= Others,____**

**3. Routes of administration: 1 = oral; 2 = intravenous; 3 = intramuscular; 4 = intravenous; 5 = subcutaneous; 6 = other,____**

**4. Purpose of treatment： 1 = Adverse events; 2 =Past/Concomitant diseases; 3 = Other,____**

**Concomitant non-Drug therapy**

| **CONCOMITANT NON-DRUG THERAPY** |
| --- |
| Did the subjects receive non-drug therapy After screening？□**_1_** Yes □**_2_** No |
| **If yes, please provide the following information：** |
| Name of the therapy：___________ |
| Frequency(if applicable)：___________ |
| Dose (if applicable) ：___________ |
| Start date：\|__\|__\|__\|__\|/\|__\|__\|/\|__\|__\| (YYYY-MM-DD)  Start time(if applicable)：\|__\|__\|：\|__\|__\|（HH：MM） |
| Ending date：\|__\|__\|__\|__\|/\|__\|__\|/\|__\|__\|(YYYY-MM-DD)  Ending time(if applicable)：\|__\|__\|：\|__\|__\|（HH：MM） |
| Still use or not：□**_1_** Yes □**_2_** No |
| Purpose of treatment：___________ |
| Other purpose of treatment, please specify：___________ |
| Indication：___________ |

**Purpose of treatment: 1 = Adverse events; 2 =Past/Concomitant diseases; 3 =Other**

**Frequency: 1=QD once daily; 2=BID twice daily; 3=TID daily 3 times; 4=QOD every other day; 5=PRN on demand; 6= Others,____**
